# Angiosarcoma of the Retroperitoneum: Report on a Patient Treated with Sunitinib

**DOI:** 10.1155/2009/360875

**Published:** 2009-05-20

**Authors:** Changhoon Yoo, Jeong-Eun Kim, Shin-Kyo Yoon, Song Cheol Kim, Jin-Hee Ahn, Tae Won Kim, Cheolwon Suh, Jae-Lyun Lee

**Affiliations:** ^1^Department of Internal medicine, Asan Medical Center, University of Ulsan, College of Medicine, 388-1 Pungnap2-dong, Songpa-gu, Seoul 138-736, South Korea; ^2^Department of Surgery, Asan Medical Center, University of Ulsan, College of Medicine, 388-1 Pungnap2-dong, Songpa-gu, Seoul 138-736, South Korea; ^3^Department of Oncology, Asan Medical Center, University of Ulsan, College of Medicine, 388-1 Pungnap2-dong, Songpa-gu, Seoul 138-736, South Korea

## Abstract

A 52 year-old woman presented with an incidentally detected retroperitoneal angiosarcoma and multiple hepatic metastases. After chemotherapy with weekly paclitaxel and doxorubicin, angiosarcoma had progressed rapidly. Because few chemotherapeutic options were available for her, sunitinib (37.5 mg/day, daily) as a salvage regimen was administered. Although sunitinib was interrupted after two weeks due to hematologic abnormalities, some metastatic nodules were regressed. Therefore, sunitinib was recommenced at a reduced dose (25 mg/day, daily). Serial computed tomography scans showed variable response in each tumor, however, sunitinib at least delayed tumor progression, compared to previous chemotherapy. With this case report, we suggest sunitinib may be effective against angiosarcomas. When sunitinib is administered to patients with angiosarcomas, hematologic abnormalities should be monitored frequently as severe hematologic toxicity may be caused either by sunitinib per se or angiosarcoma.

## 1. Introduction

Angiosarcomas are rare subtypes of soft tissue sarcomas originating from the vascular endothelium, and commonly occur in skin and soft tissues [1]. The clinical presentation of angiosarcomas is heterogeneous and multidisciplinary treatment is often necessary [2]. To treat localized disease, surgery followed by radiotherapy is a standard modality. In patients with advanced disease, paclitaxel- and doxorubicin-based regimens have been valuable [1, 2]. However, the prognosis of patients with advanced angiosarcoma remains poor, particularly when the condition progresses to a stage where cytotoxic agents are not effective [2, 3]. Today, with the development of drugs targeted to particular molecules involved in disease, several subtypes of sarcomas have been successfully treated and a number of clinical trials are ongoing [4]. Herein, we report on a patient with advanced angiosarcoma who responded favorably to sunitinib after failure of paclitaxel and doxorubicin.

## 2. Case Report

A 52 year-old woman presented with an incidentally detected retroperitoneal mass and multiple liver nodules. Her initial computed tomography (CT) and positron emission tomography (PET) scans showed a huge retroperitoneal mass with direct invasion into the spleen, pancreas tail, and stomach, and three metastatic hepatic nodules. The size of the retroperitoneal mass was 12.5 × 10 cm and the dimensions of the hepatic metastases were 4.4 × 4.1 cm in segment IV, and 2.5 × 1.9 cm and 1.2 × 1.1 cm in the right posterior segment. Percutaneous needle biopsy of a hepatic nodule was performed. In histology, irregular and sinusoidal vascular proliferation, atypical endothelial lining, and undifferentiated tumor cells were noted. These findings were consistent with angiosarcoma. This is supported by immunohistochemical study that shown tumor cells were positive for CD 31 and CD34.

**Figure 1:**
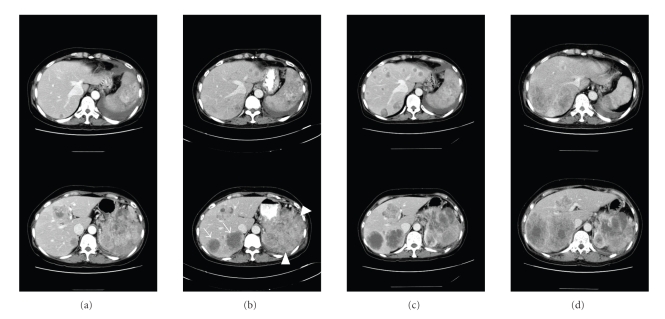
Serial CT scans show variable response in retroperitoneal mass (arrowhead), multiple nodules of left hepatic lobe and two nodules (arrows) of right posterior lobe. (a) At initial presentation, (b) after one cycle of weekly paclitaxel, (c) after two cycles of doxorubicin, (d) after 3 months of sunitinib.

Weekly paclitaxel (80 mg/m^2^, on days 1, 8, 15, and 22, every 6 weeks) was administered as first-line chemotherapy. Our patient tolerated this regime well; no significant toxicities were observed. After one cycle of paclitaxel, a CT scan (Figure 1(b)) revealed an increase in size of the three hepatic nodules and multiple new hepatic metastases. Therefore, we administered two cycles of second-line chemotherapy employing doxorubicin (60 mg/m^2^, day 1, every 3 weeks). In a follow-up CT scan (Figure 1(c)), the diameter of retroperitoneal mass was somewhat increased from 12.8 cm to 13.9 cm. However, twelve hepatic metastatic nodules were newly developed and the size of previously detected hepatic nodules was increased within two months. The disease status was rapidly deteriorated and we had few chemotherapeutic options, but our patient's general performance was sufficiently good to allow us to consider further chemotherapy. 

After a full and careful discussion with the patient, we administered sunitinib (37.5 mg/day, daily) as a salvage regimen. After 2 weeks of sunitinib, the patient presented to the clinic with severe asthenia, and chemotherapy was interrupted. A laboratory investigation revealed grade 4 thrombocytopenia, grade 3 anemia, fragmented RBCs in the peripheral blood smear, and decreased levels of both serum haptoglobin and fibrinogen. Prothombin time was within normal limits. These data supported a diagnosis of microangiopathic hemolytic anemia (MAHA). The hematologic abnormalities were suggestive of the Kasabach-Merritt phenomenon (KMP), except that coagulopathy was absent. One month later, the thrombocytopenia and anemia had regressed. At that time, a CT scan yielded interesting findings. Some of the numerous scattered hepatic nodules had disappeared, whereas others showed no change in size, and the size of the retroperitoneal mass had also decreased, with evidence of internal necrosis, although two hepatic masses in the right posterior segment had progressed slightly. When we considered the prolonged interruption to treatment caused by toxicity, and the rapid rate of progression prior to the introduction of sunitinib, we formed the view that sunitinib was active against our patient's tumors. Therefore, we recommenced sunitinib treatment, but at a reduced dose (25 mg/day, daily). Although grade 2 thrombocytopenia and MAHA reoccurred, these toxicities were less severe than before. On monthly CT scans, some hepatic metastases and the retroperitoneal mass responded to sunitinib. However, two hepatic metastases of the right posterior segment further progressed; it was thus difficult to interpret any overall chemotherapeutic effect of sunitinib. To evaluate any antitumor activity on the two hepatic masses, gross tumor volume was measured on serial CT scans (Figure 1) and the volume doubling times (VDT) between each chemotherapy regime were calculated, using the equation: VDT = [*t* × log 2]/log [*V*_*t*_/*V*_*o*_], where t is the interval in days between two scans, and *V*_*o*_ and *V*_*t*_ are tumor volumes at the previous and current examination, respectively. The hepatic mass tumors had VDT values of 16 days on paclitaxel, 66 days on doxorubicin, and 145 days on sunitinib. We thus concluded that sunitinib could delay tumor progression and the treatment was continued. 

After an additional 3 months of sunitinib therapy, recurrent hematologic toxicities associated with MAHA/KMP and concomitant deterioration of the patient's general condition caused chemotherapy to be interrupted. Two months later, the patient died of tumor invasion to her heart and inferior vena cava, about 11 months after initial diagnosis.

## 3. Discussion

Angiosarcomas are derived from vascular endothelial cells and account for less than 2% of soft tissue sarcomas [3]. The median survival time of patients with advanced angiosarcomas is about 7-8 months [2, 5]. Larger tumor size (>5 cm), location in the retroperitoneum, and advanced disease, are associated with poor prognosis [6]. Doxorubicin-based chemotherapy has been considered the standard treatment for patients with advanced angiosarcomas as well as for those with other soft tissue sarcomas [2, 6, 7]. Recent reports have shown that paclitaxel is also effective against angiosarcoma [5, 7–10]. However, few clinical drug trials on angiosarcoma patients have been carried out because of the low incidence of the disease; hence very little information on treatment is available, especially when the disease progresses after a short period of response to or stabilization with doxorubicin or paclitaxel.

Recently, tyrosine kinase inhibitors (TKIs) have emerged as novel agents in the treatment of soft tissue sarcomas [4]. There are no published clinical data on the use of TKIs as therapy for angiosarcoma patients. However, angiosarcomas originate from vascular endothelial tissues and express proangiogenic factors including VEGF [4]. These findings suggest a possible role for TKIs in angiosarcoma treatment, and several TKIs directed against angiosarcomas are currently in clinical trials [4, 11]. Sunitinib, recently approved for patients with imatinib-refractory GIST and advanced renal cell carcinoma, inhibits several tyrosine kinases and has antiangiogenic activity. The drug may thus be active against vascular endothelial cell-derived or vascular endothelial cell-dependent cancers [4, 11].

During treatment with sunitinib, our patient suffered from severe thrombocytopenia and MAHA. Although sunitinib suppresses bone marrow function, and a single case report of sunitinib-induced thrombotic thrombocytopenia purpura has appeared [12], it is difficult to conclude that the reported hematologic abnormalities were caused by sunitinib alone, because such problems have been reported in only very few GIST or renal cell carcinoma patients enrolled in large phase III clinical studies. The occurrence of severe anemia, and thrombocytopenia with laboratory features of MAHA, might be in part attributable to KMP, a syndrome associated with vascular tumors, which shows features similar to those of MAHA and consumptive coagulopathy [12–14]. 

In this case report, our patient had advanced angiosarcoma and her clinical features suggested the worst possible outcome [6]. The tumor was progressing rapidly and showed no response to conventional chemotherapy. We used sunitinib as a last resort. The overall response to sunitinib was hard to evaluate, but we observed dramatic responses in some hepatic nodules and the retroperitoneal mass, and decreased VDTs of two hepatic metastases in the right posterior segment. Eventually, her disease did progress. We suggest that individual tumor nodules may respond differently to sunitinib. Tumor nodules may gain or lose sunitinib sensitivity by operation of an unknown molecular mechanism whereas other nodules may show constitutive sunitinib sensitivity, as found in the polyclonal evolution of GIST treated with imatinib [15]. 

In conclusion, this is the first case report indicating that sunitinib may be effective against angiosarcomas. When sunitinib is administered to patients with angiosarcomas, hematologic abnormalities should be monitored frequently as severe hematologic toxicity may be caused either by sunitinib per se or angiosarcoma. Further studies are needed to explore sunitinib efficacy in angiosarcoma treatment.
